# Video-assisted Thoracoscopic surgery (VATS) lobectomy for lung cancer does not induce a procoagulant state

**DOI:** 10.1186/s12959-017-0152-2

**Published:** 2017-12-20

**Authors:** Thomas Decker Christensen, Henrik Vad, Søren Pedersen, Peter B. Licht, Mads Nybo, Kåre Hornbech, Nora Elisabeth Zois, Anne-Mette Hvas

**Affiliations:** 1 Department of Cardiothoracic and Vascular Surgery, Aarhus University Hospital, DK - 8200 Aarhus N, Denmark; 2 Department of Clinical Medicine, Aarhus University Hospital, DK - 8200 Aarhus N, Denmark; 3 Department of Anesthesiology and Intensive Care, Aarhus University Hospital, DK - 8200 Aarhus N, Denmark; 4 Department of Clinical Medicine, Aarhus University Hospital, DK - 8200 Aarhus N, Denmark; 5 Department of Cardiothoracic and Vascular Surgery, Odense University Hospital, DK - 5000 Odense C, Denmark; 6 Department of Clinical Biochemistry, Odense University Hospital, DK - 5000 Odense C, Denmark; 7 Department of Cardio-thoracic Surgery, Rigshospitalet, Copenhagen University Hospital, DK - 2100 Copenhagen, Denmark; 8 Department of Clinical Biochemistry, Rigshospitalet, Copenhagen University Hospital, DK - 2100 Copenhagen, Denmark; 9 Department of Clinical Biochemistry, Aarhus University Hospital, DK - 8200 Aarhus N, Denmark; 10 Department of Clinical Medicine, Aarhus University Hospital, DK - 8200 Aarhus N, Denmark

**Keywords:** Venous thrombosis, Blood coagulation test, Blood coagulation, Lung neoplasm, Thoracic surgery, Video assisted

## Abstract

**Background:**

Changes in the coagulation system in patients undergoing surgery for lung cancer have been sparsely investigated and the impact of the surgical trauma on the coagulation system is largely unknown in these patients. An increased knowledge could potentially improve the thromboprophylaxis regimes. The aim of this study was to assess the coagulation profile evoked in patients undergoing curative surgery by Video-Assisted Thoracoscopic Surgery **(**VATS) lobectomy for primary lung cancer.

**Methods:**

Thirty-one patients diagnosed with primary lung cancer undergoing VATS lobectomy were prospectively included. The coagulation profile was assessed preoperatively and in the first two days postoperatively using a wide range of standard coagulation tests, dynamic whole blood coagulation measured by rotational thromboelastometry (ROTEM®) and thrombin generation evaluated by calibrated automated thrombography. Patients did not receive thromboprophylactic treatment. Data was analyzed using repeated measures one-way ANOVA.

**Results:**

The standard coagulation parameters displayed only subtle changes after surgery and the ROTEM® and thrombin generation results remained largely unchanged.

**Conclusions:**

Patients undergoing VATS lobectomy are normocoagulable in the preoperative state and a VATS lobectomy does not significantly influence the coagulation.

**Trial registration:**

The trial is registered at ClinicalTrials.gov (Identifier: NCT01741506) and at EudraCTno. 2012–002409-23. Registered December 2012.

## Background

Cancer patients have an increased risk of venous thromboembolic events (VTE) consisting of deep venous thrombosis (DVT) or pulmonary embolism (PE) [1]. The risk factors for VTE are either patient-related (e.g. age, obesity), cancer-related (e.g. histopathologic type of cancer and advanced stage), treatment-related (e.g. surgical procedure, chemotherapy), or a combination of these factors [2, 3]. Patients having potentially curative operations for lung cancer differ substantially from other lung cancer patients, since they suffer predominantly from early-stage disease. Accordingly, presence of a procoagulant imbalance is not as likely as it is in patients with more advanced disease [3, 4], but has not been investigated.

The influence on the coagulation system in patients undergoing thoracic (non-cardiac) surgery has only been sparsely investigated. Specifically, serial investigations of the coagulation system in the post-operative phase are lacking. Additional knowledge is warranted, since patients undergoing surgery for lung cancer still have an increased long-term risk of VTE [1–3].

New knowledge could potentially change the thromboprophylaxis regimen, e.g. regarding type of medication (Non-vitamin K antagonist oral anticoagulant (NOAC) versus Low-Molecular-Weight Heparin (LMWH)), dose, and timing (e.g. start-up after discharge from hospital and given on a long-term basis).

Papageorgiou et al. [5] found patients with lung adenocarcinoma undergoing lobectomy to be hypercoagulable pre- and postoperatively, whereas Swiniarska et al. [6] reported that surgery resulted in an activation of the coagulation system, however significantly more profound in pneumonectomies compared to lobectomies. In a porcine model Trabjerg et al. [7] showed that there was no significant activation of the coagulation system during lung surgery. Thus, results of the published studies are conflicting and inconclusive. In order to estimate the activation of the coagulation system, advanced and validated methods should be applied. Using rotational thromboelastometry (ROTEM®), thrombin generation and standard coagulation parameters, a quantification of the coagulation system can be performed [8, 9].

We hypothesized that patients with primary lung cancer were hypercoagulable before surgery, and that the coagulation profile changes after surgery in terms of an activation of the coagulation system. The aim of this study was to assess the coagulation profile evoked in patients undergoing curative surgery by Video-Assisted Thoracoscopic Surgery (VATS) lobectomy for primary lung cancer.

## Methods

### Patient population

The present study is a sub-study to a larger randomized study already published, and the design is thus reported elsewhere [10].

Briefly, approximately 750 patients from a catchment area of 4,500,000 inhabitants are annually referred to Aarhus University Hospital, Rigshospitalet (University Hospital of Copenhagen) or Odense University Hospital, Denmark, to undergo surgery for primary lung cancer. From March 2013 to April 2015, patients who underwent lobectomy at one of these three sites were screened for eligibility.

Inclusion criteria were as follows: (1) Diagnosed with primary lung cancer with a preoperative stage IA-IB; (2) Surgery with expected lobectomy or bi-lobectomy using VATS; (3) Willingness to participate and ability to give informed oral and written consent; (4) No thromboprophylaxis received before or after surgery; and (5) > 18 years of age. Exclusion criteria were: (1) Thromboembolic event (either arterial or venous) within the past three months; (2) Pregnant or lactating; (3) Treatment with vitamin K-antagonist or a NOAC; or (4) Treatment with a platelet inhibitor if this was not paused for a minimum of 5 days (aspirin, clopidogrel or ticagrelor) or 7 days (prasugrel). No patients had received neoadjuvant chemo- and/or radiation therapy prior to surgery.

The patients were included after oral and written consent. The study protocol complied with the Helsinki II declaration and was approved by the local scientific ethical committee (File number: 1–10–72-364-12) and The Danish Data Protection Agency. The study was conducted according to Good Clinical Practice (GCP) standards and was monitored and approved by the GCP-unit, Aarhus University Hospital, Aarhus, Denmark. The trial is registered at ClinicalTrials.gov (Identifier: NCT01741506) and at EudraCTno. 2012–002409-23.

Included patients were randomized to treatment with either LMWH or no intervention. In the present study, only the subpopulation of patients receiving no intervention was included.

### Intervention

All operations were performed in general anaesthesia with propofol and fentanyl. The VATS approach used has previously been described in details [11]. Briefly, an anterior approach with one incision and two port assist incisions were performed, and one chest tube was placed. All patients were intubated with a Carlens double-lumen tube, extubated immediately after surgery and stayed approximately 12 h in the high dependency unit and then transferred back to the ward. The patients were mobilized within hours of finishing the operation and no mechanical thromboprophylaxis was provided.

### Observation period and blood analyses

Due to logistics it was not possible to perform the coagulation analyses during weekends; accordingly, only patients operated on Mondays, Tuesdays and Wednesdays were included. Accordingly, the inclusion period was somewhat elongated.

Blood samples were obtained and analyzed at the following three time-points: 1) Preoperatively; the day before surgery; 2) Postoperatively 0800 AM at day 1; and 3) Postoperatively 0800 AM at day 2.

The first 2 ml of blood was discarded before drawing blood into tubes containing sodium citrate for ROTEM® analyses, thrombin generation and standard coagulation analyses including: Activated partial thromboplastin time (APTT), International Normalized Ratio (INR), fibrinogen (functional), fibrin d-dimer, platelet count and coagulation factor VIII:Clot. Blood for ROTEM® analyses were left at room temperature for 30 min before processing, whereas the remaining analyses were done either immediately as routine analyses or blood samples were centrifuged at 2800 g for 25 min and plasma was stored in aliquots at −80 °C until analysis.

Regarding ROTEM® (Tem International GmbH, Munich, Germany), three standard assays were performed: INTEM, EXTEM, and FIBTEM. We obtained the dynamic parameters of clot initiation (clotting time: CT, seconds (s)) and clot propagation (maximum velocity of clot formation: MaxVel, mm × 100/s, time to maximum velocity: tMaxVel, s). Whole blood clot strength was assessed by maximum clot firmness (MCF, mm × 100).

Thrombin generation was evaluated by calibrated automated thrombograms (CAT; Thrombinoscope BV, Maastricht, the Netherlands) using platelet-poor plasma. The following parameters were analyzed: Lag-time until initial thrombin generation (minutes), maximum concentration of thrombin (peak, nM), time to peak (ttpeak, minutes), and the endogenous thrombin potential (ETP, nM x minutes).

The normal range for the ROTEM® was calculated based on data obtained from 73 healthy individuals previously published [12], and the normal range for thrombin generation were obtained from 90 individuals published by Vibede et al. [13].

APTT (Platelin LS, Organon, Munich, Germany), INR (Owren’s PT-reagent, MediRox), fibrinogen (Clauss method, Siemens Dade reagent) were analyzed employing the CS2100i (Sysmex, Kobe, Japan) Coagulation factor VIII:Clot (factor VIII:Clot) was analyzed by ACL-TOP (Instrumental Laboratory, Bedford, MA, USA).

Preoperatively, the following baseline analyses were performed: haemoglobin, leukocytes, platelet count, creatinine, INR and C-reactive protein (CRP).

Preoperative (baseline) data in terms of clinical characteristics was collected systematically from medical records. Furthermore, intra- and postoperative data (operating time, bleeding during surgery, transfusion, total drain loss, VTE and adverse events, length of stay and pathological staging) were registered prospectively in a case report form.

### Statistical analyses, endpoints and sample size

Baseline and postoperative characteristics were tested for normal distribution and hence presented as either mean and standard deviation (SD) or median and 95% confidence interval (CI) or as minimum to maximum values.

The results of the coagulation analyses were tested using repeated measures one-way ANOVA, sphericity was not assumed and alpha was set to 0.05. All three measurements (preoperatively, 1. postoperative day and 2. postoperative day) were needed, otherwise the patient’s measurements for that specific parameter was excluded.

Microsoft® Excel® for Mac 2011 (Microsoft®, Seattle, USA) and GraphPad Prism 6 for Mac (GraphPad Software, Inc., CA, USA) were used for the statistical analyses.

The study was explorative in nature, and a sample size calculation was therefore not performed prior to the inclusion of patients.

## Results

**Fig. 1:**
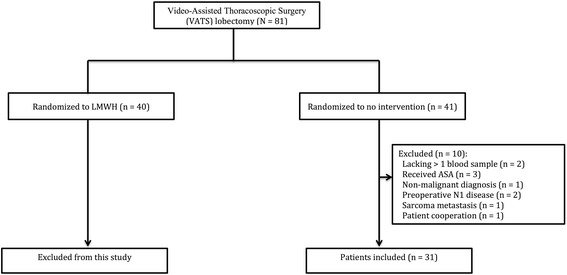
Trial flowchart for patients planned for Video-Assisted Thoracoscopic Surgery **(**VATS) lobectomy for primary lung cancer. The number of patients assessed for eligibility is not shown. Abbreviations: n/N: numbers; ASA: Acetylsalicylic acid (aspirin); LMWH: Low-Molecular-Weight Heparin; NSAID: Non Steroid Anti Inflammatory Drug

Figure 1 displays the trial flowchart. A total of 81 VATS-patients were randomized; 40 patients to the LMWH arm and 41 patients to the no intervention arm (a trial flowchart is displayed in Fig. 1). Patients randomized to the LMWH arm were excluded in the present study, and these results are published elsewhere [10].

In the no intervention arm, 10 patients were excluded (Fig. 1), and a total of 31 patients were therefore included in the present study. Among these, three patients had been given NSAID, and additionally three patients were converted to an open procedure. Three patients were operated at Odense University Hospital, three patients at Rigshospitalet (University Hospital of Copenhagen), and 25 at Aarhus University Hospital.

None of the included patients had a previously event of VTE.

Six patients (19%) received aspirin before being admitted to surgery, but all patients paused their aspirin at least 5 days prior to surgery.

Table 1 shows preoperative characteristics, while the intra- and postoperative data are displayed in Table 2. Three patients received transfusion during surgery; one patient received red blood cells, platelets and fresh frozen plasma; one patient received red blood cells and platelets and one patient received merely red blood cells.

**Table 1:** Baseline data (preoperative) of the study population prior to Video-Assisted Thoracoscopic Surgery (VATS) lobectomy for lung cancer, *n* = 31

Variables	Values
Age (years)	67 (10)
Sex (female/male), n	20/11
Non smoker/ex-smoker/ active smoker, n	1/19/11
Pack years of smoking	30 (20)
FEV 1 (% of expected)	91 (20)
DLCO (% of expected)	69 (17)
BMI	25 (4)
Co-morbidity^a^, n (%):	
Diabetes mellitus	2 (7)
Hypertension	12 (39)
Hyperlipidemia	10 (31)
Cardiac and/or vascular disease	4 (13)
Previous malignant disease	8 (26)
ASA prescribed, n (%)	6 (19)
Laboratory analyses (reference interval):	
B - Haemoglobin (women: 7.3–9.5 mmol/L; men: 8.3–10.5 mmol/L)	8.5 (0.7)
B - Leukocytes (3.5–10.0 × 10^9^/L)	7.5 (2.0)
P - Creatinine (women: 45–90 μmol/L; men: 60–105 μmol/L)	72 (18)
B - Platelet count (145–400 × 10^9^/L)	287 (85)
P - C-reactive protein (8 mg/L)	4 (4)
P - INR (< 1.2)	1.0 (0.1)
P - APTT (25–38 s)	30 (4)

*Abbreviations: B* Blood, *N/n* Numbers, *P* Plasma, *APTT* Activated partial thromboplastin time, *ASA* Acetylsalicylic acid (aspirin), *BMI* Body Mass Index, *DLCO* Diffusion Capacity of the Lung for Carbon Monoxide, *FEV1* Forced Expiratory Volume in one second, *INR* International Normalized Ratio

All values are provided either as mean and (standard deviation) or numbers and (percentage)

^a^Defined as the patient being in medical treatment for the disease

**Table 2:** Peri- and postoperative data of the study population following Video-Assisted Thoracoscopic Surgery (VATS) lobectomy for lung cancer, *n* = 31

Variables (n)	Values
Type of lobectomy (n)	
Right Upper Lobe	10
Right Middle Lobe	1
Right Lower Lobe	10
Left Upper Lobe	7
Left Lower Lobe	3
Operating time (h:m)	2:31 (0:47)
Bleeding/drainage during surgery (ml)	100 (0–2000)
Use of inotropes (patients, n)^a^	15
Re-operated	1
Total amount of fluid in the chest drain (ml)	850 (50–6135)
Complications^b^	5^c^
VTE events	0
Death	0
Total length of stay (days)	5.5 (3.2–20.0)
Type of cancer	
Adenocarcinoma	23
Squamous cell carcinoma	4
Carcinoid (all types)	1
Others^d^	3
Pathological staging	
Stage IA + B	27
Stage IIA + B	4
Microscopically free resection margins (R0)	31

*Abbreviations*: *H* Hours, *N/n* Numbers, *M* Minutes, *ml* milliliter, *VTE* Venous Thromboembolic Events

Operating time was normal distributed and is shown as mean and (standard deviation) in parenthesis, but the other data were not normally distributed and are accordingly displayed as median and (minimum to maximum)

^a^Predominantly small doses of methaoxidrin or efedrin

^b^Includes myocardial infarction, apoplexia cerebri and atrial fibrillation

^c^Apoplexia cerebri (*n* = 2) and atrial fibrillation (*n* = 3)

^d^Includes small cell carcinoma, neuroendocrine and sarcomatoid tumor

**Fig. 2:**
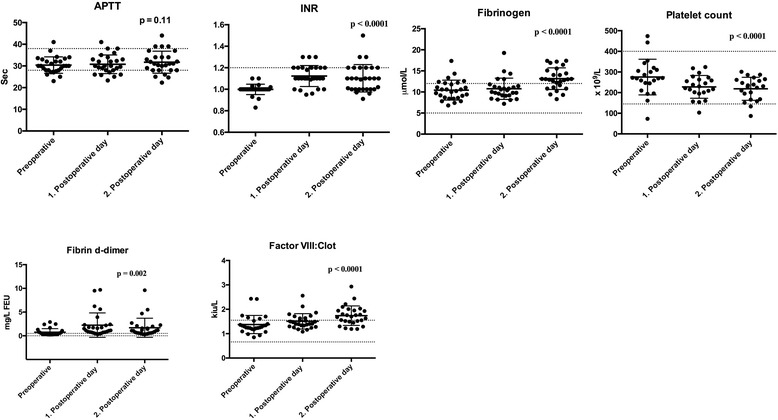
Conventional coagulation tests among 31 patients undergoing Video-Assisted Thoracoscopic Surgery (VATS) lobectomy for lung cancer. The *p* – values for the result of the ANOVA test are displayed. Values are shown as mean and standard deviation (SD), and dotted lines display the normal range (mean +/− 1.97 * SD) for healthy subjects established by the Department of Clinical Biochemistry, Aarhus University Hospital, Denmark. Abbreviations: APTT = Activated partial thromboplastin time INR = International Normalized Ratio

**Fig. 3:**
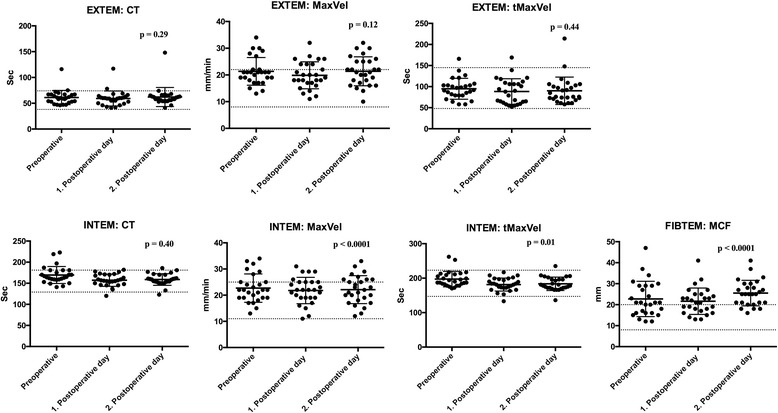
Rotem® results among 31 patients undergoing Video-Assisted Thoracoscopic Surgery (VATS) lobectomy for lung cancer. The *p* – values for the result of the ANOVA test are displayed. Values are shown as mean and standard deviation (SD), and dotted lines display the normal range (mean +/− 1.97 * SD) [12]. Abbreviations: CT = Clotting Time MaxVel = Maximum Velocity tMaxVel = Time to Maximum Velocity MCF = Maximum Clot Firmness

**Fig. 4:**
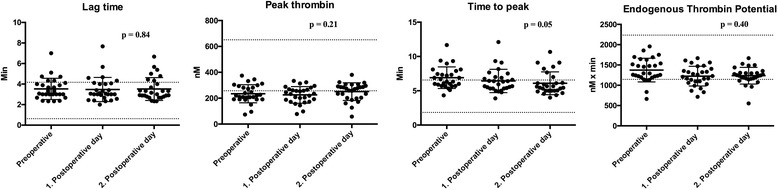
Thrombin generation among 31 patients undergoing Video-Assisted Thoracoscopic Surgery (VATS) lobectomy for lung cancer. The *p* – values for the result of the ANOVA test are displayed. Values are shown as mean and standard deviation (SD), and dotted lines display the normal range (mean +/− 1.97 * SD) [13]

The results of the ANOVA analyses regarding the standard coagulation blood tests, ROTEM® results and thrombin generation are displayed graphically in Figs. 2, 3, and 4.

The findings illustrated in Figs. 2, 3, 4 shows a statistically significant change (*p*-values are displayed in Figs. 2, 3, and 4) over time from pre- to the postoperatively state for a total of nine parameters. An increased fibrinogen, fibrin d-dimer, factor VIII:Clot and FIBTEM MCF was found reflecting a hypercoagulable state The following changes reflected a hypocoagulable state: an increase in INR, and a decrease in platelet count, INTEM tMaxVel and time to peak.

## Discussion

The main findings of the present study were that patients undergoing VATS lobectomy are normocoagulable in the preoperative state and that VATS lobectomy does not have significantly impact on coagulation. Only very few variables were found statistically significantly affected on the postoperative day 2. However, none of these changes was of clinical relevance.

This is a sub-study of a larger study, where patients undergoing curative surgery for primary lung cancer with the use of VATS were randomized to either LMWH or no thromboprophylaxis [10]. In that study, we found that the use of LMWH administered once daily as thromboprophylaxis did not alter the coagulation profile per se.

In order to investigate the temporal changes in the coagulation profiles of surgical patients, we included a group of lung cancer patients undergoing surgery receiving no thromboprophylaxis. Accordingly, we were able to follow the natural course of the coagulation system pre- and postoperatively.

The previously published studies within this area have some major limitations. The study by Trabjerg et al. [7] employed a porcine non-cancerous model, which does not necessarily reflect changes occurring in human coagulation [14]. The studies by Papageorgiou et al. [5] and Swiniarska et al. [6] had the drawback of using mainly standard blood analysis and determination of tissue factor concentration instead of global analysis as e.g. ROTEM® and thrombin generation. In contrast, the parameters used in the present study reflected the overall impact on the coagulation system [8, 9]. Moreover, it has even been advocated that thromboelastometry can be able to predict the risk of VTE after surgery [15].

The changes in the parameters fibrin d-dimer, factor VIII:Clot and FIBTEM MCF reflected a subtle hypercoagulability, while a small degree of hypocoagulability was displayed in the parameters INR, platelet count, INTEM, tMaxVel and time to peak. The absolute changes in the coagulation parameters were however small indicating that these changes are probably not of major clinical relevance.

Our patients were operated by a minimal invasive approach and VATS lobectomies are supposed to induce a reduced surgical stress compared to an open procedure [11], which probably adds to the low activation of and impact on the coagulation system. Accordingly, one can question whether this patient group benefits from thromboprophylaxis provided in the period around surgery. Further studies are obviously needed including clinical endpoints.

We chose not to include analyses of coagulation parameters during surgery, since they are influenced by several intra-operative factors such as anesthesia, surgical stress and bleeding, which introduce considerable variability. Furthermore, our aim was to investigate the changes in the coagulation profile evoked by surgery.

Of note, the present study was merely a descriptive study, since we did not perform any interventions.

Our study has some limitations because we used surrogate and not clinical endpoints in terms of VTE and bleeding events. However, the use of clinical endpoints would imply inclusion of a very large group of patients, because these events are relatively rare. Additionally, we only included blood samples up to two days after surgery and in a limited number of patients (*n* = 31).

The relatively low number of patients limits the conclusions that can be drawn from this study.

Nevertheless, we find that this fully reflects the entire intra- and postoperative period, and blood samples drawn e.g. on postoperative day 14 and day 30 would probably not have altered our conclusion, because the investigated parameters were un-altered following surgery. A larger group of patients would of course have solidified our conclusions.

We found a relatively marked variability between the individual patients at different time points for some of the ROTEM® parameters and thrombin generation variables. Even among healthy individuals the measurements in question have a relatively large variability and this fact should be taken into consideration when interpreting the results.

Besides coagulation, it would be relevant to investigate inflammatory markers in order to elucidate the cross-talk between the inflammatory and the coagulation systems. However, this was beyond the scope of this study.

Changes in coagulation are predominantly reported in adenocarcinomas, but is likely to also exist in other histopathological types [6]. However, we could have obtained more clearcut results, if we had included merely one type of cancers i.e. adenocarcinomas. Yet, the majority of cases included herein were adenocarcinomas (23 out of 31 patients corresponding to 74%).

We had a relatively high dropout rate due to the complex set-up with the blood sampling and analyses, potential interaction with medication (e.g. aspirin and NSAID) and accordingly a slow inclusion rate of patients. However, this is not assumed to have interfered with either the internal or external validity of our study.

Since all patients paused their aspirin at least 5 days prior to surgery, it is thus unlikely that aspirin administration had any impact on the conclusions drawn from this study.

The surgical procedure and technique remained the same over the entire study period. Three patients were converted to an open procedure, mainly due to bleeding, and three patients received NSAID postoperatively. We decided to include these patients in our analysis, and the inclusion did not affect the overall conclusions drawn from the study.

In order to perform a repeated measures ANOVA analyses, no missing observations were tolerated. Hence, a smaller number of patients were excluded from the statistical analysis of the respective parameters resulting in a resultant sample size of 26–29.

## Conclusions

Patients undergoing VATS lobectomy are normocoagulable in the preoperative state and a VATS lobectomy does not significantly influence the coagulation.

## Abbreviations

APTT: Activated partial thromboplastin time
CAT: Calibrated automated thrombograms
CI: Confidence interval
CRP: C-reactive protein
DVT: Deep venous thrombosis
GCP: Good Clinical Practice
INR: International Normalized Ratio
LMWH: Low-Molecular-Weight Heparin
MaxVel: maximum velocity of clot formation
MCF: Maximum clot firmness
NOAC: Non-vitamin K antagonist oral anticoagulant
NSAID: Non Steroid Anti Inflammatory Drug
PE: Pulmonary embolism
SD: Standard deviation
tMaxVel: Time to maximum velocity
VATS: Video-Assisted Thoracoscopic Surgery (VATS)
VTE: Venous thromboembolic events
